# Retrograde en bloc resection for non-muscle invasive bladder tumor can reduce the risk of seeding cancer cells into the peripheral circulation

**DOI:** 10.1186/s12957-020-1808-0

**Published:** 2020-02-10

**Authors:** Haichao Huang, Tao Wang, Metages Gashaw Ahmed, Lin Zhu, Chaoyong Yang, Wei Li, Zhun Wu, Xuegang Wang, Kaiyan Zhang, Jinchun Xing

**Affiliations:** 1 Department of Urology, The First Affiliated Hospital of Xiamen University, 55 Zhenhai Road, Siming District, Xiamen, 361003 Fujian China; 2 MOE Key Laboratory of Spectrochemical Analysis and Instrumentation, Key Laboratory of Chemical Biology of Fujian Province, State Key Laboratory of Physical Chemistry of Solid Surfaces, Collaborative Innovation Center of Chemistry for Energy Materials, Department of Chemical and Biochemical Engineering, Department of Chemical Biology, College of Chemistry and Chemical Engineering, Xiamen University, Xiamen, 361003 Fujian China

**Keywords:** Bladder tumor, Resection, Circulating tumor cells

## Abstract

**Objective:**

To ascertain whether en bloc resection could reduce the risk of seeding cancer cells into the circulation during the resection of non-muscle invasive bladder cancer (NMIBC).

**Methods:**

Patients with primary NMIBC were enrolled in this prospective study from October 2017 to May 2018. Patients were allocated to receive conventional transurethral resection of the bladder (TURB) or retrograde en bloc resection technique of the bladder tumor (RERBT). Blood samples (1 ml) for circulating tumor cell (CTC) enumeration were drawn from the peripheral vein prior to resection (PV1), immediately after resection of the tumor base (PV2), and at 12 h after resection (PV3). Intra-group comparisons of the changes in the number of CTCs identified among the PV1, PV2, and PV3 blood samples were performed in each group.

**Results:**

A total of 21 patients (12 in the RERBT group and 9 in the TURB group) were recruited. For patients receiving TURB, the level of CTCs identified in PV3 was significantly higher than that in PV1 (*p* = 0.047). However, there was no significant difference in CTC counts before and after resection in the RERBT group.

**Conclusion:**

RERBT did not increase the number of tumor cells in the bloodstream.

## Micro abstract

The aim of this study was to ascertain whether en bloc resection could reduce the risk of seeding cancer cells into the circulation during the resection of non-muscle invasive bladder cancer (NMIBC). The present study further verifies the risk of conventional TURB in promoting cell seeding during resection. Moreover, ERBT is an alternative surgical management for endoscopic treatment of NMIBC that may reduce the risk of cell seeding during resection.

## Introduction

Bladder cancer is the second most common type of urological cancer after prostate cancer [1], and transurethral resection of the bladder (TURB) is the standard in the management of non-muscle invasive bladder cancer (NMIBC) [2]. During the process of TURB, a piece-by-piece resection to the muscle layer is performed and the pressure within the bladder exceeds the venous pressure. Therefore, tumor cells may theoretically travel into the venous system during the TURB procedure. Increasing evidence shows that TURB may contribute to the increase in circulating tumor cell (CTC) count in patients with urothelial carcinoma of the bladder (UCB), and en bloc resection of the tumor may be a new approach to address this issue [3, 4].

Then, we hypothesized that en bloc resection of the bladder tumor (ERBT) but not piece-by-piece resection performed as TURB would prevent the seeding of tumor cells into the circulation during resection. To test this hypothesis, we introduced a novel retrograde en bloc resection of the bladder tumor (RERBT) technique, which has been reported in our previous study [5], and we measured the CTC counts before and after the resection. Moreover, the CTC counts before and after the resection were evaluated in patients who were treated with conventional TURB.

## Methods

### Patient and data collection

Between October 2017 and May 2018, a total of 26 consecutive patients with histologically confirmed primary UCB received transurethral resection (conventional TURB or RERBT, non-randomized) at our institution (The First Affiliated Hospital of Xiamen University). The CTC counts before and after the resection were evaluated. A preoperative flexible cystoscopy with or without biopsy was performed in all patients. To obtain a homogeneous population, the inclusion criteria were as follows: no multifocal tumors (less than 3 tumors) on cystoscopy, tumor size < 3 cm and ≥ 1 cm on cystoscopy, and no other malignancy history. Five patients were then excluded from the study, including 2 patients with squamous cell differentiation on postoperative pathological reports, 2 patients with surgery-related bleeding who received re-electrocoagulation within 24 h after the initial operation, and 1 patient with muscle invasion on the postoperative pathological report (T2 stage).

Of the 21 patients eligible for the final analysis, 9 patients received TURB, while the remaining 12 patients were treated with RERBT. Clinicopathological features, including age, gender, smoking history, tumor size and number, operation duration, and postoperative tumor grade and stage, were collected. The results of histopathology were classified according to the TNM system (the 7th edition) and graded using the WHO 2004 classification. The patients were followed up by cystoscopy every 3 months for up to 13 months of follow-up (5–13 months). Other imaging analyses, including ultrasonography, chest radiography, and abdominal CT, were performed if indicated. Recurrence was defined as intravesical recurrence at any grade and any T category. This study was reviewed and approved by the Ethics Committee of The First Affiliated Hospital of Xiamen University.

### Surgical procedure

Both RERBT and TURB were performed using the same Circom 25.6F continuous flow resectoscope with a monopolar electrode loop (Richard Wolf GmbH, Knittlingen, Germany) and with cutting and coagulation power set at 110 and 75 W, respectively (Valley Lab, USA). The fluid bag was placed 50 cm above the bladder intraoperatively and postoperatively. The administration of continuous bladder irrigation was performed for at least 12 h after the operation for all patients. All operations were performed by 3 senior urologists who had at least 5 years of experience in endoscopic treatment of NMIBC (KYZ, WL, and ZW). The final decision on the modality of surgery (TURB vs. RERBT) was reached using a surgeon/patient decision prior to surgery but not a randomization.

The process of the RERBT technique was introduced briefly as follows (Additional file 1: Video S1). Blood vessels entering the tumor were blocked before resection by electro-coagulating the macroscopic normal mucosa approximately 0.5 to 1.0 cm away from the tumor base to reduce intraoperative hemorrhaging. During the resection, bleeding vessels were coagulated simultaneously, providing a better visualization. Therefore, the time and degree of vascular opening was reduced and the opportunity for tumor cells to enter the bloodstream was kept to a minimum. Most importantly, the tumors were removed en bloc resection in RERBT rather than a piece-by-piece resection as in TURB [5]; there was no tumor chip floating in the bladder during the resection, and continuous bladder irrigation was performed after the resection.


**Additional file 1: Video S1.** A patient with a 1.6-cm-diameter lesion underwent RERBT.


### CTC isolation and detection

Blood samples (1 ml) for CTC enumeration were drawn from the peripheral vein prior to resection (PV1), immediately after resection of the tumor base (PV2), and at 12 h after resection (PV3). Meanwhile, a total of four volunteers (2 males, 2 females) who had no history of malignant tumors were recruited for CTC analysis as a control group. Evacuated EDTA-coated blood collection tubes were used to collect blood samples and then kept at 4 °C before enumeration. All samples were analyzed within one working day.

**Fig. 1:**
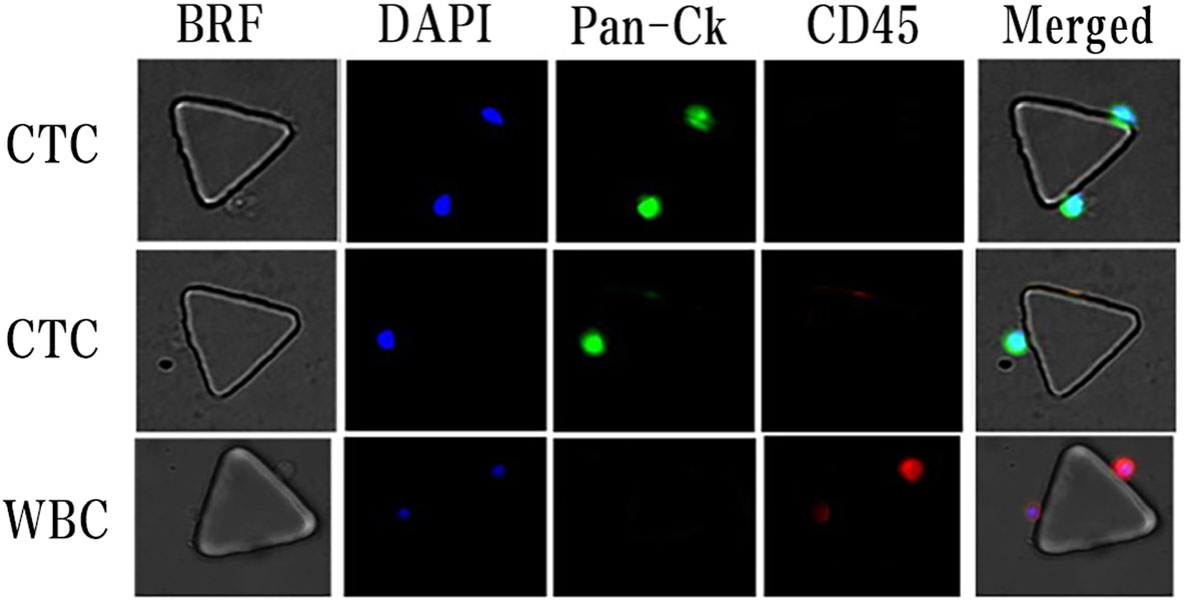
A CTC is defined as a nucleated cell (DAPI-positive) with CK-positive and CD45-negative phenotype, and a white blood cell is defined as CD45-negative

We employed a new device, a size-dictated immunocapture chip (SDI-Chip) [6], to measure the CTC counts. Epithelial cell adhesion molecule (EpCAM) and cytokeratin (CK) were used as positive stains, lymphocyte common antigen (CD45) as a negative stain, and 4′,6-diamidino-2-phenylindole (DAPI) as a nuclear stain to distinguish tumor cells from leukocytes. With this assay, a CTC was defined as a nucleated cell (DAPI-positive) with CK-positive and CD45-negative phenotype (Fig. 1). Enumeration of CTCs was performed by two experienced laboratory researchers (LZ and AMG) who were blinded to the clinicopathological data. In the event of any discrepancies, the researchers re-evaluated the images together until a consensus was reached.

### Statistical analysis

For statistical analysis, numerical variables were compared with *t* tests and categorical variables were compared with the chi-square test (Fisher exact test) using Statistic Package for the Social Sciences (SPSS) software, version 22.0. In addition, intra-group comparisons of the changes in CTC counts among the PV1, PV2, and PV3 time points were performed by paired *t* tests. In all tests, a two-tailed *p* value of < 0.05 was considered to indicate significance.

## Results

### Baseline characteristics and survival outcomes

**Fig. 2:**
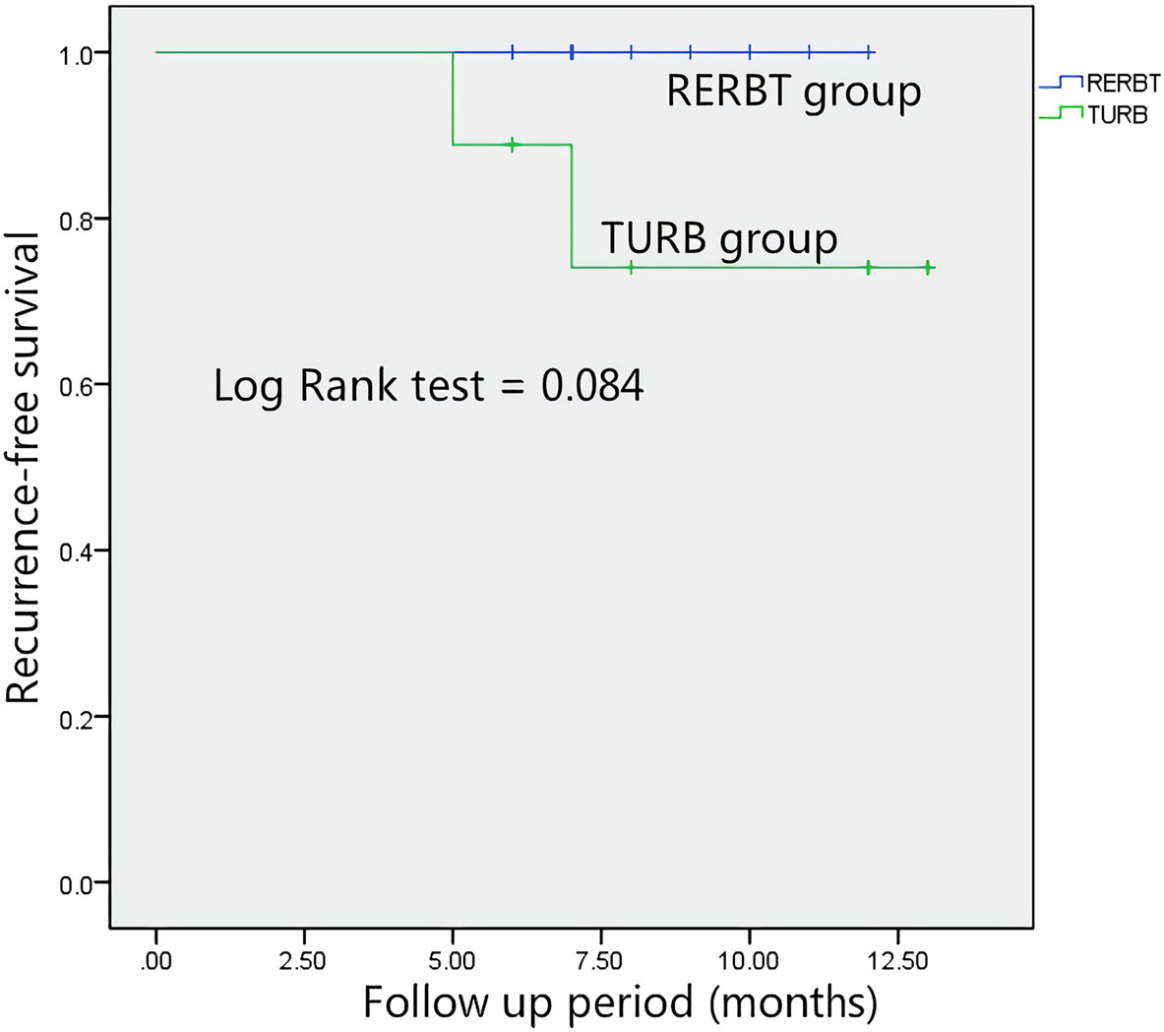
Recurrence-free rate according to transurethral resection methods. Conventional TURB (group 1; green line) and RERBT (group 2; blue line). All patients (*n* = 21) were analyzed using the log-rank test

A total of 21 patients with primary NMIBC were involved in our study; 9 patients were treated with TURB, while the remaining 12 patients received RERBT. There were no statistically significant differences in terms of demographics, smoking history, operation duration, postoperative tumor grade or stage (Table 1), and baseline CTC counts (Table 2) between the two groups. Until November 2018, 2 patients experienced disease recurrence, with a median follow-up period of 8 months (5–13 months). Both patients who experienced disease recurrence were in the TURB group. Survival data displayed in Kaplan–Meier curves demonstrated no statistically significant differences with respect to recurrence-free survival (RFS) between the TURB and RERBT groups (*p* = 0.084, Fig. 2).

**Table 1:** Descriptive clinicopathological characteristics of non-muscle invasive bladder cancer patients treated with TURB and RERBT

Characteristics	TURB, *n* (%) *N* = 9	RERBT, *n* (%) *N* = 12	Total	*p* value
Age (year)				0.670
< 60	4 (44.4)	7 (58.3)	11	
≥ 60	5 (55.6)	5 (41.7)	10	
Gender				1.000
Male	7 (77.8)	9 (75.0)	16	
Female	2 (22.2)	3 (25.0)	5	
Smoking history				0.660
No	3 (33.3)	6 (50.0)	9	
Yes	6 (66.7)	6 (50.0)	12	
Pathologic stage				0.660
pTa	6 (66.7)	6 (50.0)	12	
pT1	3 (33.3)	6 (50.0)	9	
Size (mean, cm)	1.59 ± 0.68	1.88 ± 0.59	21	0.301
Grade (WHO 2014)				1.000
LG	7 (77.8)	10 (83.3)	17	
HG	2 (22.2)	2 (16.7)	4	
Operation duration (min)				1.000
< 60	6 (66.7)	7 (58.3)	13	
≥ 60	3 (33.3)	5 (41.7)	8	
Operation duration (mean, min)	45.44 ± 15.14	47.83 ± 14.90	21	0.722
Total	9 (42.9)	12 (57.1)	21	

*TURB* transurethral resection of the bladder, *RERBT* retrograde en bloc TURB, *LG* low grade, *HG* high grade

**Table 2:** Correlation between preoperative CTC counts and clinicopathological factors

Characteristics	*n* (%)	CTC ($\overline{\mathrm{x}}\pm \mathrm{s}/\mathrm{ml}$)	*t*	*p* value
Age (year)			− 0.213	0.833
< 60	11 (52.4)	5.64 ± 7.31		
≥ 60	10 (47.6)	6.40 ± 9.08		
Gender			− 2.687	0.019
Male	16 (76.2)	3.57 ± 4.97		
Female	5 (33.8)	0.00 ± 0.00		
Smoking history			− 1.735	0.099
No	9 (42.9)	2.67 ± 5.66		
Yes	12 (51.1)	8.50 ± 8.79		
Pathologic stage			0.764	0.454
pTa	12 (51.1)	7.17 ± 7.31		
pT1	9 (42.9)	4.44 ± 9.04		
Grade (WHO 2014)			− 2.497	0.022
LG	17 (81.0)	4.12 ± 6.42		
HG	4 (19.0)	14.00 ± 10.07		
Resection method			− 1.350	0.193
TURB	9 (42.9)	3.33 ± 5.83		
RERBT	12 (51.1)	8.00 ± 9.03		

*LG* low grade, *HG* high grade, *CTC* circulating cancer cell, *TURB* transurethral resection of the bladder, *RERBT* retrograde en bloc TURB

### Correlation between preoperative CTC counts and clinicopathological factors

There were 16 males and 5 females in this study, and the male gender was significantly associated with higher preoperative CTC counts compared with the association with the female gender (*p* = 0.019). A similar association was observed between tumor grade and preoperative CTC counts (*n* = 4.12 ± 6.42/ml for patients with low-grade NMIBC vs. *n* = 14.00 ± 10.07/ml for patients with high-grade NMIBC, *p* = 0.022). Preoperative CTC counts were not associated with age, smoking history, or tumor stage (*p* = 0.833, 0.099, and 0.454, respectively) (Table 2).

### Changes in CTCs before and after tumor resection

To further verify whether TURB may lead to an increase in CTCs in the bloodstream, blood samples were drawn from the peripheral vein before resection (PV1), after resection of the tumor base (PV2), and at 12 h after resection (PV3). The mean CTCs at PV1, PV2, and PV3 in the TURB group were 3.33 ± 5.83/ml, 11.78 ± 11.85/ml, and 22.67 ± 24.25/ml, respectively. Compared with PV1, a significantly higher number of CTCs was observed at PV3 (*p* = 0.047). Although the PV2 sample did not show a significant increase in CTCs compared to PV1, and PV3 did not show a significant increase in CTCs compared to PV2 (*p* = 0.056 and 0.068, respectively), there was an insignificant increasing trend of CTC number during the entire TURB process.

On the other hand, the mean CTCs of PV1, PV2, and PV3 in the RERBT group were 8.00 ± 9.03/ml, 8.00 ± 9.80/ml, and 4.33 ± 5.52/ml, respectively. There were no significant changes in CTCs among the PV1, PV2, and PV3 samples in the RERBT group (the *p* values of PV3 vs. PV1, PV2 vs. PV1, and PV3 vs. PV2 were 0.176, 1.000, and 0.094, respectively). In addition, for the four control volunteers, no measurable CTCs were observed.

## Discussion

NMIBCs are known as malignant urothelial tumors that do not invade the detrusor and are staged as Ta, T1, or carcinoma in situ (CIS). TURB remains the gold standard for the management of NMIBC. However, the piece-by-piece resection pattern violates the basic oncological surgical principles of en bloc resection and may lead to tumor cell dispersal. Despite complete resection, these tumors have a high propensity to recur and progress, with unacceptable rates of recurrence and progression as high as 78 and 45% within 5 years after the initial TURB, respectively [7]. Does the TURB itself contribute to the poor oncological outcomes for NMIBC, which is traditionally categorized as superficial bladder cancer?

In a recent preliminary study including 17 UCB patients, Engilbertsson and colleagues reported that TURB could cause the seeding of tumor cells into the bloodstream during the process of TURB [3]. This finding has since been validated by another prospective study. In this study, Blaschke et al. showed that the number of CTCs was increased postoperatively in 3 of 8 patients who received TURB for UCB [4]. In our study, 9 patients received conventional TURB for NMIBC, and the CTC counts 12 h after the resection were significantly higher than the CTC counts before the resection in the bloodstream (*p* = 0.047), which was consistent with the results of the two studies mentioned above [3, 4]. The underlying mechanisms may lie in the fact that (1) tumors are resected separately in fractions but not resected via en bloc during traditional TURB; (2) the integrity of the bladder wall is damaged; and (3) the pressure within the bladder exceeds the venous pressure during the process of TURB. Taken together, conventional TURB violates the basic oncological surgical tenet of en bloc resection, promoting the spread of the tumor debris and cells into the systemic circulation through damaged vascular walls during the piece-by-piece resection. Further development of novel TURB techniques is needed to overcome these disadvantages. ERBT represents an alternative technique for endoscopic treatment of NMIBC. For tumors of more than 1 cm in size, ERBT could remove the whole tumor in a single piece [8], and it is available to retrieve whole tumor specimens up to 4.5 cm in size [9]. ERBT has the potential to overcome tumor fragmentation and possible consequences including cell seeding and low-qualified specimens, which are the biggest limitations of conventional TURB.

To test whether ERBT could reduce the risk of cell seeding, we introduced a novel RERBT technique [5] and compared the number of CTCs identified before and after resection using a size-dictated immunocapture chip, which had been reported in our previous study [6]. In the current study, all the tumors were less than 3 cm in size, so we were able to retrieve the tumor in one piece when ERBT was performed. As a result, there was no tumor debris during the resection process. After ERBT, tumor cells may have less chance to remain in the bladder and further insert into the circulation during irrigation. In contrast, when conventional TURB was performed, tumor cells are more inclined to remain in the bladder after resection, leading to a further increase in the number of CTCs. Moreover, timely electrocoagulation of the bleeding vessels was performed due to better visualization during the ERBT, which may contribute to a decrease in the risk of cell seeding through damaged vascular walls. Thus, there was no significant difference in the changes in CTCs identified among the PV1, PV2, and PV3 time points in patients who were treated with RERBT, which was in contrast with the results of patients treated with conventional TURB, where a significant increase in the CTC counts was observed.

Although whether ERBT, compared with conventional TURB, could provide a better long-term survival for patients with NMIBC is controversial, a recent meta-analysis in which patients who received ERBT showed a significantly lower 24-month recurrence rate than that of patients who received conventional TURB [10]. In our study, 2 patients who experienced disease recurrence were in the TURB group. During the follow-up period, no patients in the RERBT group experienced disease recurrence. However, there were no significant differences with respect to RFS between the TURB and RERBT groups (*p* = 0.084), which may be due to the relatively shorter survival period. Incomplete resection, cell implantation, or tumor biology itself may contribute to these results in terms of recurrence rate. Moreover, in a recent, small prospective study, the progression rate, though without significance, was lower in patients who received ERBT than in patients who received conventional TURB [11]. We suggested that the improvement in terms of the progression rate may be due to the reduction in cell seeding during the process of resection when ERBT was performed. Based on a series of studies, the presence of CTCs has been confirmed to be associated with a variety of solid malignancies, including breast, colorectal, gastric, and prostate cancer [12–15]. Although inconsistent results with regard to the association between the clinicopathological features and the presence of CTCs in patients with UCB have been reported [16–21], the association between the detection of CTCs and poor oncological outcomes of UCB patients has already been confirmed by a variety of studies [18–20, 22]. Recently, a systemic review study regarding the association between CTC-positive results and the clinicopathological features and prognostic outcomes in 2161 patients with UCB showed that the presence of CTCs in the peripheral blood was an independent predictive indicator of both poor histopathological parameters and poor oncological outcomes [23]. Furthermore, in the first report regarding the presence of CTCs in NMIBC patients using CellSearch, Gazzaniga et al. showed that the detection of CTCs was significantly associated with both poor histopathological parameters and poor oncological outcomes [18]. Similar results were also observed in a recent study by Gradilone and colleagues. In this study, which included 54 histologically confirmed T1G3 UCB patients, the detection of CTCs in the peripheral blood circulation was an independent risk factor for poor disease-free survival [22]. Similarly, in our study, patients with histopathologically confirmed high-grade NMIBC had a significantly higher level of preoperative CTCs than did patients with low-grade NMIBC (*p* = 0.022). Therefore, CTCs play an important role in the survival of UCB patients. However, whether the difference in the changes in CTCs during tumor resection contributes to a higher progression rate in patients who received conventional TURB than in patients who received ERBT was unclear, as it is an extremely complex process from cell seeding to micrometastasis and distant metastasis. Further studies are needed to validate this hypothesis in the future.

There are two main limitations of this study. First, it is not a randomized study. However, we enrolled a homogeneous population, and there was no significant difference between RERBT and conventional TURB groups in the distribution of clinicopathological features, including tumor grade which was associated with the presence of CTCs. Moreover, there was no significant difference in the preoperative CTC counts between the TURB group and the RERBT group (*p* = 0.193). The difference in changes in CTC counts before and after resection was evaluated using an intra-group comparison. The results of one group would not affect the results of the other group. Second, the limited sample size was also a limitation of our preliminary study. As the cost of CTC enumeration once was almost $1000 in China, so at least $3000 was required for one patient in our study. CTCs are unlike other simple and inexpensive biomarkers that can be evaluated on a large scale without considering the cost-effect in a preliminary study. We believe that this cost-effect issue has been taken into consideration in studies from western populations [3, 4]. There are still several limitations of our study. First, all resections were performed by three surgeons. Second, we enrolled patients without multifocal NMIBC in the present study. In addition, the tumors enrolled were sized as < 3 cm and ≥ 1 cm on cystoscopy. Thus, whether RERBT remains a prophylactic effect for multifocal tumors or tumors of a larger size is unclear. However, to the best of our knowledge, we are the first to demonstrate that RERBT did not increase the number of cells in the bloodstream compared to conventional TURB, which may be a potential reason to explain the better oncological outcomes of patients who received ERBT than the outcomes of patients who were treated with conventional TURB [10, 11]. We believe our preliminary study will encourage other groups conducting further prospective studies with larger sample sizes to gain insight into this field. Moreover, we hope our preliminary study will support other groups who are committed to improving the transurethral resection techniques and upholding oncological principles in the endoscopic management of bladder tumors.

## Conclusion

The present study further verifies the risk of conventional TURB in promoting cell seeding during resection. Moreover, ERBT is an alternative surgical management for endoscopic treatment of NMIBC that may reduce the risk of cell seeding during resection. To our knowledge, these results are novel.

## Data Availability

Research data are not shared.
